# p53 Modulation of Autophagy Signaling in Cancer Therapies: Perspectives Mechanism and Therapeutic Targets

**DOI:** 10.3389/fcell.2022.761080

**Published:** 2022-01-26

**Authors:** Md Ataur Rahman, Moon Nyeo Park, MD Hasanur Rahman, Md Mamunur Rashid, Rokibul Islam, Md Jamal Uddin, Md Abdul Hannan, Bonglee Kim

**Affiliations:** ^1^ Department of Pathology, College of Korean Medicine, Kyung Hee University, Seoul, South Korea; ^2^ Korean Medicine-Based Drug Repositioning Cancer Research Center, College of Korean Medicine, Kyung Hee University, Seoul, South Korea; ^3^ Global Biotechnology & Biomedical Research Network (GBBRN), Department of Biotechnology and Genetic Engineering, Faculty of Biological Sciences, Islamic University, Kushtia, Bangladesh; ^4^ Department of Biotechnology and Genetic Engineering, Bangabandhu Sheikh Mujibur Rahman Science and Technology University, Gopalganj, Bangladesh; ^5^ ABEx Bio-Research Center, Dhaka, Bangladesh; ^6^ Department of Oncology, Lombardi Comprehensive Cancer Center, Georgetown University Medical Center, Washington, DC, United States; ^7^ Department of Biotechnology and Genetic Engineering, Faculty of Biological Sciences, Islamic University, Kushtia, Bangladesh; ^8^ Department of Biochemistry, College of Medicine, Hallym University, Chuncheon, South Korea; ^9^ Graduate School of Pharmaceutical Sciences, College of Pharmacy, Ewha Womans University, Seoul, Korea; ^10^ Department of Biochemistry and Molecular Biology, Bangladesh Agricultural University, Mymensingh, Bangladesh

**Keywords:** p53, autophagy, apoptosis, tumor suppressor, synthetic drug, phytochemical

## Abstract

The key tumor suppressor protein p53, additionally known as p53, represents an attractive target for the development and management of anti-cancer therapies. p53 has been implicated as a tumor suppressor protein that has multiple aspects of biological function comprising energy metabolism, cell cycle arrest, apoptosis, growth and differentiation, senescence, oxidative stress, angiogenesis, and cancer biology. Autophagy, a cellular self-defense system, is an evolutionarily conserved catabolic process involved in various physiological processes that maintain cellular homeostasis. Numerous studies have found that p53 modulates autophagy, although the relationship between p53 and autophagy is relatively complex and not well understood. Recently, several experimental studies have been reported that p53 can act both an inhibitor and an activator of autophagy which depend on its cellular localization as well as its mode of action. Emerging evidences have been suggested that the dual role of p53 which suppresses and stimulates autophagy in various cencer cells. It has been found that p53 suppression and activation are important to modulate autophagy for tumor promotion and cancer treatment. On the other hand, activation of autophagy by p53 has been recommended as a protective function of p53. Therefore, elucidation of the new functions of p53 and autophagy could contribute to the development of novel therapeutic approaches in cancer biology. However, the underlying molecular mechanisms of p53 and autophagy shows reciprocal functional interaction that is a major importance for cancer treatment and manegement. Additionally, several synthetic drugs and phytochemicals have been targeted to modulate p53 signaling via regulation of autophagy pathway in cancer cells. This review emphasizes the current perspectives and the role of p53 as the main regulator of autophagy-mediated novel therapeutic approaches against cancer treatment and managements.

## Introduction

Autophagy, a self-degradative intracellular process, is an essential mechanism of the cell that facilitates renewal or removal of cellular molecules, thereby balancing the cell’s energy consumption and maintaining homeostasis (Rahman and Rhim, 2017; Rahman et al., 2020a). However, autophagy deregulation is now considered to be one of the most characteristic features for tumor progression (White, 2015). It has recently been revealed that autophagy suppression and a combination of chemotherapeutic treatment have been approached as a potential treatment for cancer (Perez-Hernandez et al., 2019), although this depends on the context and type of cancer. To date, numerous tumor suppressor oncogenes and proteins have emerged as eminent autophagy regulators whose mutation or depletion regulates autophagy as well as tumorigenesis. Evidences have been suggested that p53 which belonging to the tumor suppressor genes may act as an inhibitor or activator of autophagy depending on their mode of action and subcellular localization (Lacroix et al., 2020). Morevoer, physiological role of autophagy in cancer offers a highest possible target for future cancer therapy and is, hence, presently intensively investigated. Therefore, understanding p53 regulation and its role in individual cellular contexts with a suitable approach of autophagy-mediated regulation in cancer is crucial for drugs development that might be targeted autophagy in a specific diseases model.

Tumor suppressor p53 has been implicated in a wide variety of cellular processes, including genomic stability, cell-cycle arrest, DNA repair, apoptosis, cellular senescence, and autophagy (Aubrey et al., 2018; Mrakovcic and Frohlich, 2018). Generally, p53 binds to DNA in the nucleus which regulates transcription of target genes to activate apoptosis (Tang et al., 2021b). Nevertheless, human p53 mutation has been encouraged tumor progression, chemoresistance, and apoptosis (Alvarado-Ortiz et al., 2021). Additionally, p53 inactivation is effectively used as a therapeutic target of a promising approach to trigger anti-cancer therapy (Zawacka-Pankau and Selivanova, 2015). Thus, p53 has a dual role as a positive or negative regulator of autophagy in cancer (Liu and Gu, 2021). Under normal cellular conditions, p53 has been recognized as an autophagy inhibitor, while in response to stress or starvation, p53 might be translocated into the nucleus which endorsed autophagy via transactivation with its target genes (Mrakovcic and Frohlich, 2018; Fang et al., 2021). p53 functions have been modulated via several post-translational modifications as well as different interacting proteins (Soussi, 2000). Among them, 14-3-3 family proteins play an important function in p53 regulation in response to DNA damage (Falcicchio et al., 2020). However, reasons for this difference in wild-type and mutant p53 activities have been triggered apoptosis and cell cycle arrest remain unclear (Parrales and Iwakuma, 2015). Particularly, experimental studies have been confirmed that mutant with gain-of-function variant of p53 in tumors cells are characterized via a higher genomic instability in response to reduce chemotherapeutic which has usually poor prognosis for patients (Liu et al., 2012). In this review, the molecular mechanisms and regulation of autophagy in cancer would be discussed regarding modulation of p53. Additionally, recent progress of autophagy signaling in tumor microenvironment in addition to its targeting for possible cancer therapeutics developments from the pre-clinical trials anong with the challenges in developing autophagy-based cancer therapy (Mukhopadhyay et al., 2021). Therefore, current approaches triggering p53-mediated autophagy regulation in cancer treatment are highlighted and summarized in cancer cells to conventional treatments which are able to overcome chemoresistance in cancer.

## Methods

Literature-based online databases, Google Scholar, Web of Science, PubMed, Google, and Scopus were accessed to collect information on the published articles that reported molecular mechanism of p53 and autophagy modulation in cancer prevention. Several keywords were used in the search, such as p53, autophagy, cancer, phytochemicals, natural compounds, solid tumors, and lymphomas perspectives role of p53 and autophagy in cancer therapy. Figures were created with the Adobe Illustrator software.

## Biological Function of p53 Signaling in Cancer

**FIGURE 1:**
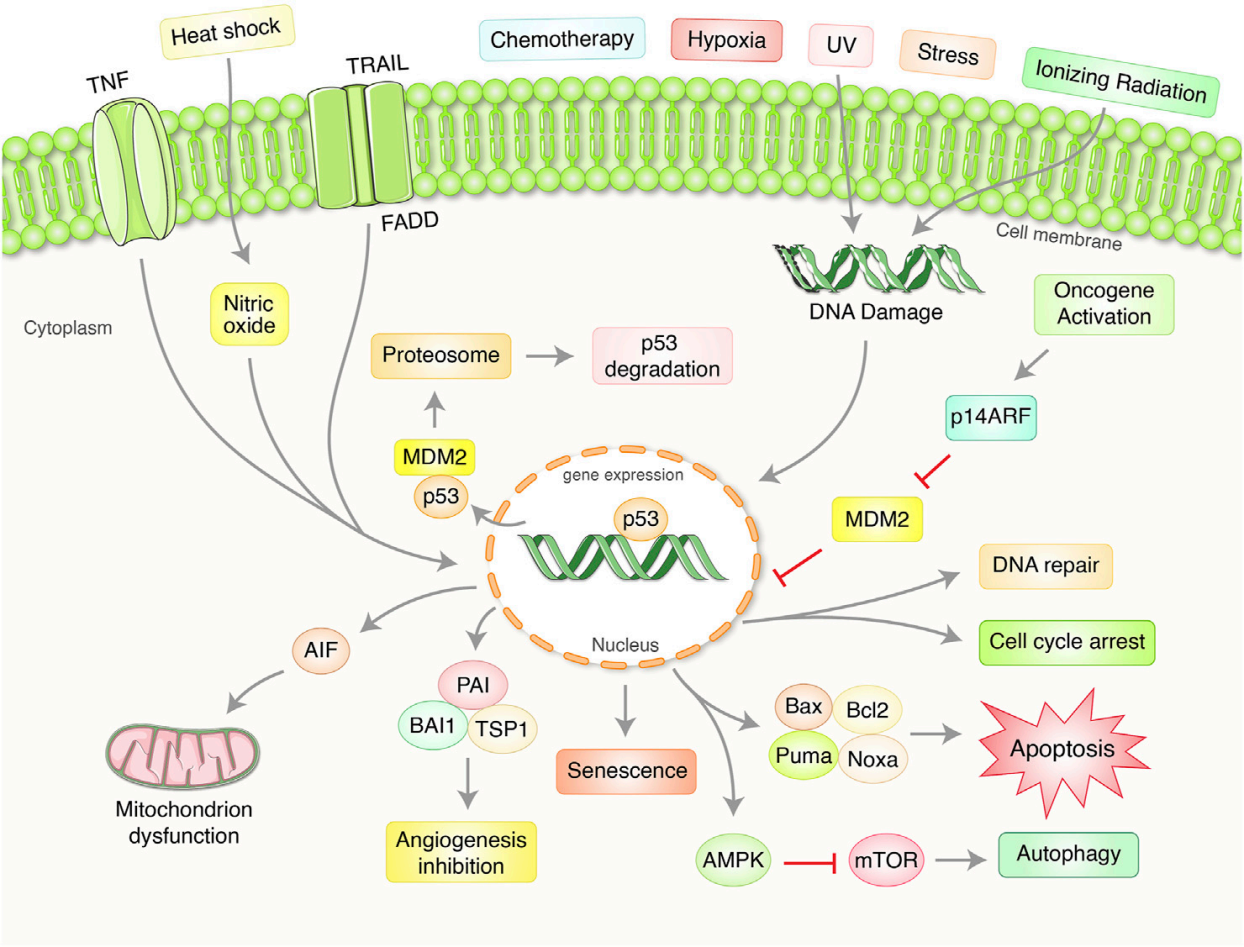
Importance and regulation of tumor suppressor p53 pathway in the regulation of cancer. p53 protein plays an essential role in coordinating with a complex signaling network which regulate aberrant cell proliferation and growth. Normally, p53 has preserved at low steady-state levels with crucial regulation of two proteins, murine double minute 2 (MDM2) as well as MDMX. MDM2 mediates an attachment of ubiquitin (Ub)-mediated proteasomal degradation. Exposure to ultraviolet (UV) light and ionizing radiation activate several kinases and damaging stressors. Oncogenes overexpression has been found to stimulate the production of alternative reading frame (ARF), p14ARF in human and p19ARF in mouse, which binds to MDM2 as well as stabilizes p53. Activation of p53 protein has been targeted to transactivate numerous gene expressions depending on the stressors and the cell type which significantly control either DNA repair, cell cycle arrest, senescence, apoptosis, mitochondrion regulation, autophagy, and angiogenesis.

The p53 is a central transcription factor that has the capacity to induce diverse cellular responses likely DNA damage repair, cell cycle arrest, apoptosis, and senescence followed by various stress signals (Figure 1) (Subburayan et al., 2018; Mijit et al., 2020). The master biological function of p53 is to ensure the safety of the DNA uprightness of the cell (Munroe et al., 2020). Along with this, p53 protein operates some additional acts in cellular aging, cell differentiation, and development (Jain and Barton, 2018). The p53 antitumor function is broadly governed by dual approaches; it can promote repair of the DNA damage or promote apoptosis or autophagy to completely remove the irreplaceable damaged materials or cells (Crighton et al., 2006; Janicke et al., 2008). In fact, p53 is a transcription factor of the nucleus which governs the diverse array of cellular processes and escorts transcription of a broad group of target genes of it. At the initial phase of DNA damage, p53 activates and induce cell-cycle arrest of G1-phage which is attributed to repair the DNA damage by promoting the transcription of p21WAF1, GADD45, and p53R2 (He et al., 2020). Following the DNA repair, cells can start come back into the regular cell cycle procedure resulting in p53 itself regulate nuclear integrity to prohibit tumor induction or occurrence (Williams and Schumacher, 2016; Cafaro et al., 2020). On the other hand, p53 is able to apply its pro-apoptotic activities through the removal of the damaged cells (Ingaramo et al., 2018). In this cellular process, p53 is responsible for the transactivation of a wide range of pro-apoptotic target genes that encodes Bax, Bak, Puma, and Noxa proteins belong to BH-3 only protein and playing a role to promote apoptosis in a cell (Moll et al., 2006; Labi et al., 2008) (Figure 1). In this manner, p53 can protect from tumorigenesis or cancer initiation by regulating this complex process.

### Role of Mutant p53 Contributes to Autophagy Regulation in Cancer

It has been found that mutant p53 proteins are involved in different autophagic pathways vai degrading and targeting to explore the potential approaches in cancer through autophagy (Shim et al., 2021). p53 mutant has designated as a gain-of-oncogenic function(s) (GOFs) which improved cell migration, proliferation, as well as invasion with anti-apoptotic functions which dynamically contribute to numerous phases of tumor progression in cancer (Dittmer et al., 1993; Oren and Rotter, 2010). The changes beyond cancerous are subjected to deliberate discriminating benefits such as facilitating angiogenesis, continuous growth avoiding growth signal, insensitivity to cancer drugs, promotes adequate metabolism, escape from apoptosis with the self-sufficiency of stress signal and ultimately promoting metastasize and invasion (Chatterjee and Viswanathan, 2021; Hernandez Borrero and El-Deiry, 2021). Furthermore, growing evidences from *in vitro* and *in vivo* have signified that the oncogenic activities of p53 mutant variants have heterogeneous which can vary with tissue type in addition to genetic background of the cells (Eriksson et al., 2017). Almost 50% of the p53 gene is mutated in cancer cells, which underlying its normal role in cancer suppression, favors interchange or inactivate the gene which gains a new function that cooperates to sustain the abnormal growth of cancer (Boutelle and Attardi, 2021). Additionally, mutant p53 proteins have been found to exert on autophagy while other mutant p53 activities might affect diverse aspects of cancer biology. It was found that ectopically overexpressing 22 different p53 mutant variants control autophagy in p53 null colon cancer cells (Morselli et al., 2008). p53^R175H^ or p53^R273H^ mutants suppresses autophagic vesicles formation and lysosomes fusion via the transcriptional suppression of p53 key downstream responsive autophagy related protein such as DRAM1, BECN1, ATG12, SESN1/2, P-AMPK, and TSC2 (Cordani et al., 2016). Furthermore, protein-protein interactions with other transcription factors as a GOF and some cancer-associated p53 mutants have been shown the capability to block autophagy indirectly via triggering numerous growth factor receptors as EGFR, TGFBR, and IGFR which contributing to sustain PI3K/Akt/mTOR signaling and subsequently suppress autophagy in cancer (Aschauer and Muller, 2016). Therefore, targeting of p53 mutant proteins by autophagy inhibition and activation might offer a promising future therapeutic opportunity and is thus presently investigated intensively to modulate autophagy in cancer therapies.

## Biological Function of Autophagy in Cancer

**FIGURE 2:**
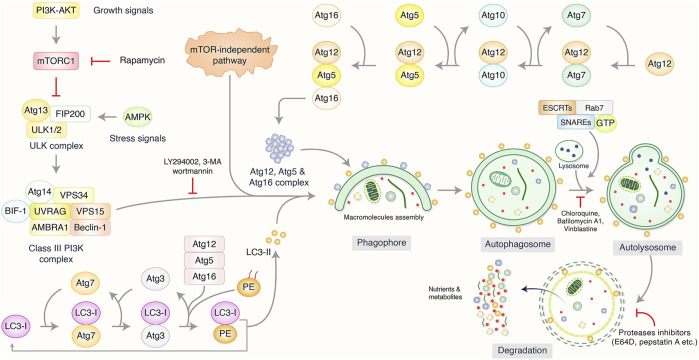
Biological function and molecular mechanism of autophagy pathway. Autophagy has been initiated by the formation of pre-autophagosome structure via the action of several proteins. PI3K-AKT and mammalian target of rapamycin (mTOR) have been influenced to initiate pre-autophagosome assembly via association of ULK1/VPS34/Beclin-1 complex. Additionally, Atg5/Atg12/Atg16 and Atg12/Atg5/LC3 complexes are involved to create phagophore nucleation and macromolecules accumulation which has been elongated as well as bind to autophagosome formation. Lysosome binds mature autophagosome by the help of ESCRT/SNARE/Rab7 protein complex, resulting in autolysosome formation. Finally, autolysosomes have been abolished by acid hydrolases resulting in the release of recycling metabolites as well as nutrients.

Autophagy has been categorized as an intracellular self-degradation mechanism through dysfunctional cytoplasmic organelles and aggregated misfolded proteins are terminated via fusion with lysosomes and double-membrane autophagosomes to maintain cellular homeostasis (Krishnan et al., 2020; Miller and Thorburn, 2021). Usually, autophagy process, mainly macroautophagy, has been initiated via the isolation of pre-autophagosome structures called phagophore assembly sites (PAS) (Hurley and Young, 2017). PI3K related to the endoplasmic reticulum (ER) have a vital role to initiate PAS formation (Kotani et al., 2018). Unc-51 like autophagy activating kinase-1 (ULK1), mammalian target of rapamycin (mTOR), and AMP-activated protein kinase (AMPK) facilitate phagophore formation during induction of autophagy (Alers et al., 2012; Rahman et al., 2021a). Nevertheless, VPS34/UVRAG/Beclin-1/AMBRA1 helps in the phagophore formation (Velazquez and Jackson, 2018), followed by membrane elongation and autophagosome formation (Rubinsztein et al., 2012). Lysosome binds to mature autophagosome by the association of ESCRT/SNARE/Rab7 protein complex, resulting in the formation of autolysosomes (Kardideh et al., 2019; Rahman et al., 2021c). Finally, autolysosomes that contain misfolded/aggregated proteins have been degraded via acid hydrolases and provide recycling metabolites and nutrients for maintaining intracellular homeostasis (Figure 2). It has been found that cancer cell fate regulations and development depended on the autophagy process (Wei and Huang, 2019).

Additionally, well-known cellular autophagy mechanism contributing to carcinogenesis is chaperone-mediated autophagy (CMA) which signify lysosomal-mediated degradation process to facilitate cell survival (Chava et al., 2017). It has been found that during serum starvation, CMA and macroautophagy are triggered consecutively signifying that these two paths are not entirely independent while deficiency or blockage one of this pathway may lead to activate other (Kaushik et al., 2008; Cuervo and Wong, 2014). However, CMA has been found to degrade mutant p53 in a lysosome-dependent fashion in cancer cells under nonproliferating conditions (Vakifahmetoglu-Norberg et al., 2016). Later, chaperone-assisted selective autophagy (CASA) was found in skeletal muscle cells which coordinates protein synthesis and degradation and act as an important physiological stimulus crucial for cellular development, respiratory, maintain urogenital systems, and homeostasis of locomotory (Ulbricht et al., 2013). Moreover, CASA machinery ensures proteostasis in addition to regulate essential cellular developments such as proliferation, migration, and adhesion which comprises the molecular chaperones HscA8/Hsp70 as well as HspB8/Hsp22 alone with the co-chaperones Bag3 and STUB1/CHIP (Liu et al., 2013). Importantly, it is found that CASA is essential for muscle maintenance (Arndt et al., 2010). Therefore, HscA8/Hsp70 and HspB8/Hsp22 compex and CASA play an significant function in protein quality control of cancer cells.

Accumulating evidence indicated that autophagy could decide whether cancer cells are promoted or suppressed in certain conditions (Rahman et al., 2020a). In that case, mTOR has an essential function either cellular function becomes an oncogenic activating or protective via inactivation or induction of autophagy pathway (Uddin M. S. et al., 2020; Rahman et al., 2021b). In addition, chemotherapeutic drugs were shown to suppress tumor cells by autophagic modulation (Rahman et al., 2020b). Also, autophagy inhibition has been regulated in cancer progression which decides whether autophagy influences cell death or cell survival function (Jung et al., 2020). Furthermore, epigenetic and genetic function might be alternated the Atgs gene expression which has a greater impact on cancer cell survival. Thus, autophagy modulation of cancer cells has been found to examine the distribution of tumor microenvironment progression which contributes to the potential management and prevention of cancer (Rahman et al., 2020b). Therefore, p53 may react to different kinds of stress as well as damage employed on the cell which comprise endogenous- or environmentally-stressed genotoxicity, oxidative stress, and oncogene activation in order to protect cell damage as well as maintain cellular integrity in cancer (Liu and Gu, 2021). Nevertheless, how posttranslational modifications of p53 postulate its selectivity for each of these transcriptional targets as well as the particular cellular function which induce autophagy in cancer is still unclear.

## p53 Signaling Targets as a Cancer Therapy via Modulation of Autophagy

**FIGURE 3:**
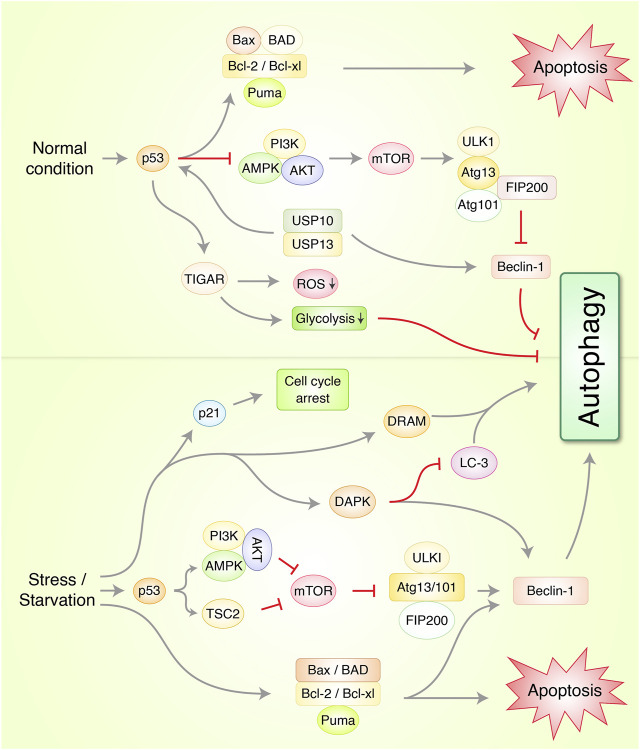
Molecular mechanism of autophagy and apoptosis via p53 regulation in cancer under normal and stress conditions. In normal condition, p53 protein prevents autophagy-mediated cell death via induction of Beclin-1 degradation through ubiquitin-specific peptidases USP10/USP13 and AMPK/mTOR/ULK1 complex activation. TP53-induced glycolysis and apoptosis regulator (TIGAR) prevents autophagy through suppression of reactive oxygen species (ROS) and glycolysis formation. Under stress/starvation condition, p53 activates AMPK and tuberous sclerosis complex 2 (TSC2) which suppresses mTOR and ULK1/FIP200 complex which finally stimulates autophagy. Cyclin-dependent kinase inhibitor 1, p21, activates and arrests cell cycle via p53-mediated upregulation. Additionally, death-associated protein kinase (DAPK), and damage-regulated autophagy modulator (DRAM), autophagy-related protein Beclin-1 upregulation initiates autophagy. Bcl-2 family, Bcl-2, Bcl-xl, Bax, Bad, and PUMA activates apoptosis.

Cell death regulation is a complicated process of maintaining cellular homeostasis by preventing oncogenic growth and recycling damaged cell debris (Rangel et al., 2021). Dysregulation of autophagic cell death occurs frequently in a variety of malignancies and poses a barrier to current therapy development Rahman et al., 2020. Autophagy plays a critical role in both tumor promotion and suppression. Autophagosomes engulf and digest cell organelles and proteins, which are then recycled to restore homeostasis and cellular metabolism (Duffy et al., 2015). In recent years, it has been proposed that the suppression of autophagy in combination with chemotherapy could be used as an innovative way to treat cancer (Figure 3). Interference with the autophagic machinery, on the other hand, can promote or disrupt carcinogenesis, depending on the type of cancer and their environment. It is, therefore, critical to uncover the primary signaling mechanisms that control carcinogenesis and regulate autophagy (Mrakovcic and Frohlich, 2018). Recently, it has been found that autophagy enhanced the stemness of lung CSCs via degrading ubiquitinated p53, therefore relieving cytosolic p53 inhibition of autophagy through generating stable human lung CSC cell lines of wild-type TP53 (A549) where TP53 has been deleted (H1229) (Wang J. et al., 2021).

The research to date has found several tumor suppressor proteins and oncogenes to be essential regulators of autophagy. The loss or mutation of these proteins contributes to tumor formation. In addition to being one of these tumor suppressors, the mammalian cell “janitor” p53 may be one of the most frequently mutated genes in human tumors. Most human cancers exhibit p53 mutation, which is found in approximately half of all tumors (Soussi and Wiman, 2007; Shi Y. et al., 2020). P53 activation is dependent on various stressors, such as DNA damaging agents, oncogenes, and hypoxia, as well as others, and leads to changes in cell cycling, apoptosis, senescence, metabolism, differentiation, as well as angiogenesis inhibition and autophagy control (Giaccia and Kastan, 1998; Levine and Abrams, 2008). From the results of recent experimental research, it has been ascertained that p53 has both an activator and an inhibitor function with regard to autophagy, depending on its cellular localization and the way of operations (Mrakovcic and Frohlich, 2018). p53 may play a pro-autophagic role in the nucleus, both in a transcription-dependent and independent manner. In the cytoplasm, on the other hand, p53 is known to suppress the induction of autophagy (Maiuri et al., 2010). Recently, it has been highlighted that interplay between pro-inflammatory/pro-oncogenic and pro-inflammatory cytokines pathways regulated via UPR signaling as well as autophagy which affects the stability of p53 that is able to control UPR signaling, cytokine release, and autophagy to preserve its own stability in additional to promote tumorigenesis against cancers carrying mutp53 (D'Orazi et al., 2021).

There are many different cell stressors that might activate p53 (Punja et al., 2021). It is possible that activated p53 may downregulate the autophagy negative regulator, mTOR, through transcriptional regulation of Sestrin1 and Sestrin2, which activate AMPK, which then phosphorylates tuberous sclerosis 2 protein (TSC2) (Budanov and Karin, 2008; Maiuri et al., 2009). In addition to AMPKβ1 and AMPKβ2, p53 can transactivate other AMPKβ subunits including TSC2, PTEN, and IGF-BP3. All of these AMPKβ subunits can be upregulated in response to a stress signal, and upon this elevation, the p53-dependent negative regulation of the mTOR pathways takes place (Feng et al., 2007; Eby et al., 2010). All of these AMPK subunits are capable of being upregulated in response to a stress signal, and this upregulation results in the p53-dependent negative regulation of the mTOR pathways (Jazvinscak Jembrek et al., 2021). A number of targeted genes are activated or inhibited by p53, suggesting that autophagy and cancer prevention are achieved through p53 actions (e.g., activating AMPK and inhibiting mTOR). DRAM (damage-regulated autophagy modulator), a p53 target gene encoding a lysosomal protein that induces macroautophagy, is another mechanism by which p53 promotes the activation of the autophagic pathway. In addition, AEN/ISG20L1 was found to modulate autophagy in response to genotoxic stress by interacting with members of the p53 family (Eby et al., 2010). The three p53 family members (p53, p63, and p73) can regulate transcription of AEN, and downregulation of AEN expression results in decreased levels of autophagic vacuoles and LC3-II, which indicates genotoxic stress. In addition to positive regulators of autophagy, several other pro-apoptotic genes such as PUMA (p53-upregulated modulator of apoptosis) and Bax (Bcl-2-associated X protein) act as autophagy stimulators. It has been discovered that the protein PUMA, which is only found in the mitochondria, induces mitochondrial autophagy. This function of PUMA is distinct from the function of autophagy induced by starvation or ER stress, which is dependent on the presence of the Bax or Bak proteins. Additionally, mitochondrial-selective autophagy can be induced in the absence of PUMA activation in the presence of only Bax (Yee et al., 2009). These pro-apoptotic genes are likely to induce apoptosis and autophagy in a manner that is closely related. Through its direct physical interaction with the BCL-xL receptor, the p53-regulated tumor suppressor protein p14ARF (alternate reading frame protein product of the CDKN2A locus) appears to be able to induce autophagy in human cancer cells (Pimkina et al., 2009; Balaburski et al., 2010). It has been recently confirmed that p14ARF’s tumor suppressive properties are achieved through autophagy activation (Verma et al., 2021). Additionally, the same report resolved previous discrepancies between two p14ARF mRNA isoforms and demonstrated that autophagy can only be induced by the full-length p14ARF mRNA in the nucleus, while mitophagy is induced by smARF (selective macroautophagy of mitochondria) (Ueda et al., 2008; Budina-Kolomets et al., 2013). Studies have found that p53-mediated autophagy begins with DAPK-1 stimulation, with increased gene expression as a secondary response (Zalckvar et al., 2009b). In order to carry out autophagy, DAPK-1 uses two different routes. In the one instance, Beclin-1 phosphorylation inhibits the BCL-2/BCL-xL-mediated degradation of Beclin-1, while in the other, LC3-interacting MAP1B inhibition keeps autophagy from proceeding (Harrison et al., 2008; Zalckvar et al., 2009a).

It has been reported that in p53^−/−^ cells, only the cytoplasmic p53 can inhibit autophagy through suppressing AMPK and inducing mTOR, resulting in the hyperphosphorylation of AMPK, TSC2, and acetyl CoA carboxylase (ACC) and hypophosphorylation of mTOR substrate, p70S6K (Tasdemir et al., 2008a). Autophagy in HCT116p53^−/−^colon carcinoma cells is reduced when they are re-transfected with the p53 wild-type allele. Furthermore, when transfected into p53^−/−^ cells, p53 mutants that preferentially localize to the cytoplasm are found to effectively repress autophagy (Morselli et al., 2008). According to all of these observations, it is evident that p53 in the cytoplasm inhibits autophagy. It has been previously shown that TIGAR (TP53-induced glycolysis and apoptosis regulator) has a molecular link to p53’s anti-autophagic function (Bensaad et al., 2006). Under stressful conditions, inhibition of autophagy by TIGAR, which is a direct target gene of the tumor suppressor gene p53, has been shown to be associated with downregulation of glycolysis and suppression of ROS formation (Bensaad et al., 2009). When TIGAR’s function is impaired, ROS levels increase, triggering autophagy induction. Nevertheless, it is unlikely to have an effect on the mTOR pathway (Tang et al., 2021a). It is most likely to have a non-mTOR-mediated metabolic pathway as TIGAR does not appear to have a significant impact on mTOR signaling. The interaction of p53 in embryonic carcinoma cells with Beclin-1 leads to the ubiquitination and degradation of the p53, which thus suppresses autophagy (Tripathi et al., 2014). By inhibiting cytoplasmic p53, this effect can be reversed, and autophagy can be induced more effectively.

Cancer cells acquire unique metabolic characteristics to ensure their survival and proliferation (DeBerardinis, 2008). Recent studies have been shown that p53 regulates metabolic traits of cells in addition to its role as a tumor suppressor protein (Wen and Wang, 2021), but the exact mechanism by which p53 regulates metabolism is still not completely understood. As a compensatory response to protect cells against stress, increased signaling triggered by p53 leads to activation of the PtdIns3K-Akt-MAPK-Ras signaling pathway (Corcoran et al., 2006). It was suggested by Gottlieb and Vousden that p53 might be able to counteract the Warburg effect, which is characterized by an abnormally high rate of glycolysis under aerobic conditions and is seen in many cancers (Gottlieb and Vousden, 2010). Recent studies have concluded that p53-regulated metabolism and autophagy are linked which is a primary strategy for cancer treatment to manipulate autophagy regulated by the p53 gene (Shim et al., 2021). A study conducted by Buzzai *et al.* examined the effect of the anti-diabetic drug metformin on tumor growth in the presence of metformin in the colon cancer cell lines HCT116 p53^+/+^ and HCT116p53^−/−^, which were isogenic colon cancer cell lines. Autophagy was discovered to be activated in the presence of metformin in HCT116 p53^+/+^ cells but not in HCT116 p53^−/−^ cells in the presence of metformin, which contributed to the continued survival of the cells both *in vitro* and *in vivo* (Buzzai et al., 2007; Sui et al., 2011).

## Therapeutic Aspect of p53 Pathway Modulation of Autophagy in Cancer

Recently, numerous p53-targeting treatment strategies have been established which includings dendritic cell-derived vaccines, adenoviral p53 vectors, p53-degrading E3 ubiquitin ligase inhibitors of Mdm2, and small-molecules to reinstate DNA binding activity. For example, a small molecule multi kinase inhibitor, sunitinib, has been permitted to treat metastatic renal cell carcinoma which degrade autophagic induction of wild type p53 proteins in a multiple cancer cell lines (Luo et al., 2018). Additionally, several synthetic and naturally occurring molecules have been targeted to regulate p53-mediated autophagy regulation in cancer. There are several newly discovered drugs and phytochemicals used as MDM2 inhibitors that have shown potential p53-mediated cancer preventive activities *in vitro* and *in vivo*. This section will focus on their efficacy and mechanisms of action.

### Synthetic Drug Targeting p53-Mediated Autophagy Modulation in Cancer

**FIGURE 4:**
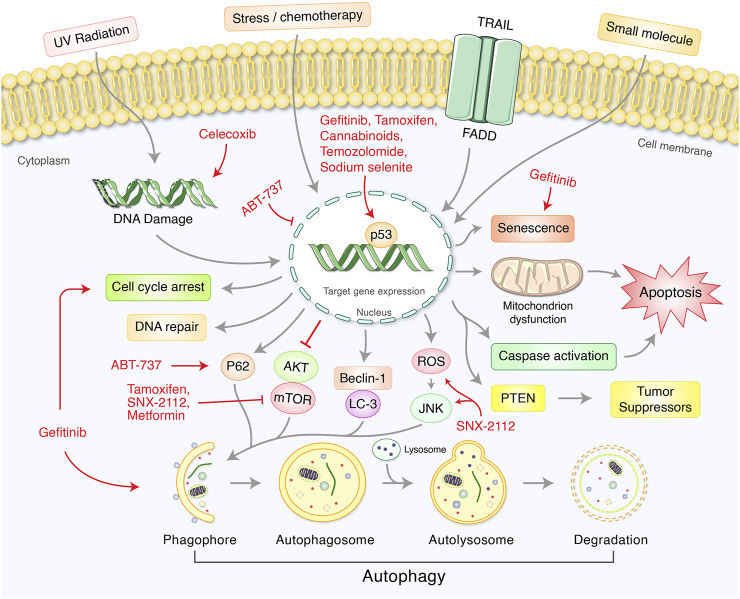
Synthetic drugs targets for p53-mediated autophagy modulation in cancer therapy.

Several synthetic chemicals have been used to modulate p53-mediated regulation of autophagy signaling in cancer treatment (Table 1, Figure 4). Synthetic cannabinoids was used to induce mitochondrial-mediated apoptotic and autophagy pathways in human LN18, T98G, and U251MG glioblastoma cells deficient in TP53 or PTEN tumor suppressors (Ellert-Miklaszewska et al., 2021). Gefitinib has been found to improve disease outcomes in non-small cell lung cancer (NSCLC) patients via activation of autophagy, apoptosis, senescence, and cell cycle arrest through augmenting the expression of LC3B-II, cleaved caspase-3, p21, and p53 (Zhu et al., 2015). BH3 mimetic, ABT-737, induced autophagy related protein LC-III and decreased P53 in HCT116 colon carcinoma cell lines (Tasdemir et al., 2008b). In HepG2 liver cancer cell, ABT-737 increased p62, Beclin-1, and p53 (Du et al., 2013). COX-2 inhibitor celecoxib-induced DNA damage, activated p53-dependent G-1 cell cycle arrest and regulated p53-dependent autophagy induction in human glioblastoma cells (Kang et al., 2009). Tamoxifen, a first line adjuvant endocrine therapy, was increased peptidylarginine deiminase 2 (PAD2), nuclear p53, cell cycle arrest, and apoptosis via downregulating Akt/mTOR expression in tamoxifen-resistant MCF-7 (MCF7/TamR) cells (Li et al., 2019). In contrast, Hsp90 inhibitor SNX-2112 enhanced cellular apoptosis via ROS-mediated autophagy pathway in human cervical cancer cells (Hu et al., 2019). Temozolomide has been found to induce autophagy and p53 as well as phospho-p53 levels in glioblastoma U87 cells (Lee et al., 2015). Moreover, sodium selenite induced autophagy and apoptosis in p53 wild type cells without caspase-8/apoptosis-inducing factor activation and upregulated PLSCR1 in Leukemia NB4 cells (Shi K. et al., 2020). It has been reported that anti-diabetic drug, metformin, activated autophagy via mTOR inhibition and AMPK activation in p53-deficient tumor cell growth of cancer HCT116 p53^+/+^ and HCT116 p53^−/−^ cell lines (Buzzai et al., 2007). Furthermore, metformin inhibited matrix metalloproteinase-9 activation, decreased endogenous insulin resistance, suppressed HER2 (erbB-2) oncoprotein overexpression, improved cancer patient’s survival in type 2 diabetes, and blocked migration as well as invasion of cancer cells (Sui et al., 2011). Recently, Saini *et al.* found that verteporfin, known as autophagy inhibitory and proteotoxic functions, disrupts multiple steps of autophagy in addition to regulate p53 to sensitize osteosarcoma of human osteosarcoma cells- HOS (R156P mutant P53) (Saini et al., 2021). microRNA, miR-26b, improves the sensitivity of hepatocellular carcinoma to doxorubicin by USP9X-dependent degradation of p53 as well as autophagy regulation (Chen et al., 2021).

**TABLE 1:** Several therapeutic drugs targeting p53-mediated autophagy regulation in cancer therapy.

Serial	Drugs	Model/Cancer type	Mechanism of p53 modulation	Autophagic condition	References
1	Synthetic cannabinoids	Human LN18, T98G, and U251MG glioblastoma cells	Mudulation of mutant p53	Inducetion of autophagy	Ellert-Miklaszewska et al. (2021)
2	Gefitinib	Non-small cell lung cancer (NSCLC)	Increases p53 expression	Autophagy activation	Zhu et al. (2015)
3	ABT737	HCT116 colon carcinoma cell	p53 induction	Autophagy activation	Tasdemir et al. (2008b)
4	ABT737	HepG2 liver cancer cell	Activation of p53	Autophagy induction	Du et al. (2013)
5	Celecoxib	Human glioblastoma cells U87MG and LN229 cells	p53 modulation	Induction of autophagy	Kang et al. (2009)
6	Tamoxifen	MCF-7 (MCF7/TamR) cells	Activate nuclear p53	Induction of autophagy	Li et al. (2019)
7	SNX-2112	Cervical cancer cells (HeLa cells)	p53 induction	Activates autophagy	Hu et al. (2019)
8	Temozolomide	Glioblastoma U87 cells	Modulate p53	Induction of autophagy	Lee et al. (2015)
9	Sodium selenite	Leukemia NB4 cells	Wild type p53 Modulation	Induction of autophagy	Shi et al. (2020a)
10	Metformin	HCT116 p53+/+ and p53−/− Colon cancer cell	Mudulation of p53-deficient tumor cell	Activatation of autophagy	Buzzai et al. (2007)
11	Verteporfin	Human osteosarcoma cells- HOS	p53 ubiquitinated proteins modulation	Autophagy inhibition	Saini et al. (2021)
12	Doxorubicin	Human HCC cells (HepG2, Hep3B) SNU387, and SNU449	Modulation of p53 de-ubiquitination	Autophagy regulation	Chen et al. (2021)

### Phytochemicals/Natural Products Targeting p53-Mediated Autophagy Regulation in Cancer Therapy

**FIGURE 5:**
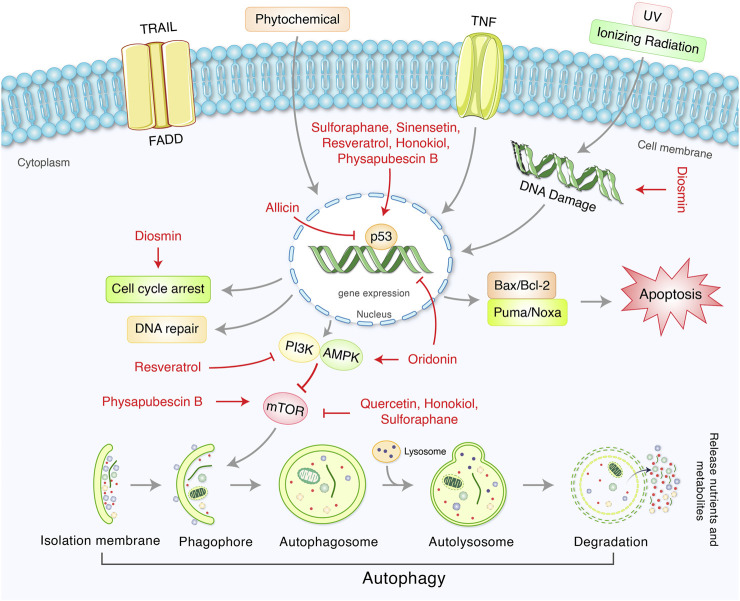
Therapeutic actions of phytochemicals and their targeted signaling system in p53-mediated autophagy regulation in cancer.

Phytochemicals from edible as well as medicinal plants have shown to potent cancer chemotherapeutic and chemopreventive activities. Several phytochemicals have mediated their anticancer properties via targeting p53 (Qin et al., 2018) (Figure 5). Numerous phytochemicals/natural products have been used to modulate p53-mediated autophagy pathways as a therapeutic target are presented in Table 2. Allicin reduced cytoplasmic p53, Bcl-2, and inhibited PI3K/mTOR signaling pathway in addition to increase AMPK/TSC2 and Beclin-1 expression in Hep G2 cells (Chu et al., 2012). Sinensetin-mediated autophagy has been involved in p53-induced AMPK/mTOR signaling pathway in HepG2 Cells (Kim et al., 2020). In p53 wild, HCT116 cells, luteolin exhibited anti-cancer effects via the regulation of p53 through cell cycle arrests such as PARP/p21 and apoptosis mediated by Nova and Bax (Yoo et al., 2021). Quercetin, a flavonoid derived from fruits and vegetables, was found to induce p53-independent/mTORC1 mechanism in various cancer cells such as human hepatocellular carcinoma cells (HepG2, Hep3B, MDA-MB-231) and colorectal cancer cells (HCT116, GFP-LC3 Hela cells) (Wang Z. X. et al., 2021). It has been demonstrated that resveratrol inhibited pAkt/Akt and induced autophagy related protein Beclin-1, LC3-II and p62 in HCC cells (Zhang et al., 2018). The anticancer mechanism of mimulone has been mediated by an increase of specific markers of autophagy such as LC3-I and LC3-II along with inhibition of p53, *p*-mTOR and increase of *p*-AMPK (An et al., 2014). Diosmin, derived from citrus fruits, has been identified as a mediator of oxidative and nitrosative stress caused by DNA damage and DNA methylation lead to G2/M cell cycle arrest, elevation in p53, p21, p27 and ERK, mediated by autophagy (Lewinska et al., 2017). Honokiol, a lignan belonging to the genus *Magnolia*, induced ROS-mediated autophagic cell death via regulating the p53/PI3K/Akt/mTOR signaling pathway in human U87 MG glioma cells (Lin et al., 2016). Oridonin, a natural diterpenoid isolated from the traditional Chinese herb, activated autophagy through inhibition of glucose metabolism and AMPK inhibition in p53-mutated colorectal cancer cell (Yao et al., 2017). A steroidal compound, physapubescin B, extracted from *Physalis pubescens* L. (Solanaceae), has been described to possess anti-cancer potential through excessive ROS generation and induce p53-dependent apoptotic cell death by autophagy inhibition in cervical cancer (HeLa) and colon cancer (HCT116) cells (Xu et al., 2017). Sulforaphane (SFN), an isothiocyanate compound found in cruciferous vegetables, potentiates apoptosis and promotes autophagy in malignant mesothelioma cells via activation of p53 (Lee and Lee, 2017; Uddin MS. et al., 2020). A steroidal saponin, A-24, derived from *Allium chinense*, induced apoptosis and autophagy along with migration inhibition in p53 wild-type as well as p53-deficient gastric cancer cells via ROS accumulation in independent of p53 (Xu et al., 2021).

**TABLE 2:** Numerous phytochemicals used as a therapeutic target of p53-mediated autophagy modulation in cancer.

Sl	Phytochemicals	Model/Cancer type	Mechanism of action	p53 Condition	Autophagic condition	References
1	Allicin	Hep G2 liver cancer	AMPK/mTOR/TSC2 activation	p53 level decreased	Autophagy induction.	Chu et al. (2012)
2	Sinensetin	Hep G2 human liver cancer	AMPK/mTOR. inhibition	p53 modulation	Increases autophagy	Kim et al. (2020)
3	Luteolin	HCT116. HT-29 colon cancer	Apoptosis activation	p53 level increased	Autophagy Induction	Yoo et al. (2021)
4	Quercetin	HepG2, Hep3B, MDA-MB-231, HCT116	Activation of apoptosis, TFEB, cathepsin B, cathepsin D, and LAMP-1	p53 level increased	Autophagy induction.	Wang et al. (2021b)
5	Resveratrol	HCC human hepatocellular carcinoma cells	PI3K/Akt and Beclin1, LC3 II, and p62 activation	p53 level increased	Autophagy induction.	Zhang et al. (2018)
6	Mimulone	Human A549, MCF-7, HCT116, U2OS cells	AMPK/mTOR activation	p53 level decreased	Induction of autophagy	An et al. (2014)
7	Diosmin	MCF-7, MDA-MB-231, SK-BR-3 cell	p21, p27, and ERK1/2 activation	p53 level increased	Autophagy induction.	Lewinska et al. (2017)
8	Honokiol	Human U87 MG glioma cells	Akt/mTOR downregulation	p53 induction	Autophagy induction	Lin et al. (2016)
9	Oridonin	HCT-15, COLO205, HCT116, RKO, SW480, and SW620	AMPK deactivated autophagy induction	p53 decresed	Induction of autophagy	Yao et al. (2017)
10	Physapubescin B	HeLa and HCT116	mTORC1 and ROS suppression	p53-dependent	Autophagy inhibition	Xu et al. (2017)
11	Sulforaphane	Malignant mesothelioma (H-28)	Akt/mTOR reduction	p53 level increased	Induction of autophagy	Lee and Lee, (2017)
12	A-24	p53 wild-type and-deficient gastric cancer cells	PI3K/Akt/mTOR pathway	p53 modulation	Autophagy induction	Xu et al. (2021)

### Perspectives and Limitations of p53-Modulated Autophagy Cancer Therapy

The role of p53 in autophagy regulation in cancer progression has established into a strongly knit, exciting, and rapidly changing disciple in biological science. However, the study of the ability of p53 to modulate autophagy in addition how this modulation of regulation of cancer metabolism raises numerous issues. The basic process of autophagy is important for normal cellular function as its dysregulation is generally encountered during human tumor development (Yan and Chen, 2021). However, p53 and autophagy comprise a two-edged sword as well as possess an important function in tumor development and progression (Thorburn, 2014; Gao et al., 2020). Depending on the cancer type and entity, p53 and autophagy molecular predisposition in relation to tumor mutations, both can either encourage or inhibit tumorigenesis (Mrakovcic and Frohlich, 2018). There is currently not much evidence of p53-mediated autophagy regulation in cancer metabolism. Recently, it has been found that p53 activates cell cycle arrest in MEFs cell, whereas it induces apoptosis in oncogene-transformed MEFs cell which indicates that p53 exerts its tumor inhibition function in a cell- and tissue-dependent manner (Kon et al., 2021). Meanwhile, autophagy activation leads to clearance of subcellular organelle, or autophagic cell atrophy, or autophagic cell death in which tumor suppression occurs upon activation of p53 in a certain type of tissue (Jin, 2005). Additionally, autophagy activation contributes to determining cell fate upon p53 activation (Chen, 2016). However, autophagy downregulation either via mutations of autophagic genes, or activation of mTOR signaling through the activation of an abnormal oncogene might change p53-mediated apoptosis or necrosis with cell cycle arrest (Denisenko et al., 2018). Forthcoming studies would be required to investigate the epigenetic and genetic modifications of autophagy pathway in cancer in the context of p53 tumor suppression. p53 network and mTOR network will not only provide a new understanding of tumorigenesis, but also provide a clue for the target of cancer chemotherapy. Accordingly, the function of normal p53 might be compromised. Furthermore, the consequences of autophagy regulation via p53 modulation for cancer prognosis are quite difficult to predict. The link between p53 and autophagy provide a novel mechanism which p53 might play an important functional role as a guardian of metabolic balance in cancer suppression. These new functional role of p53-mediated autophagy modulation would be provided an interesting potentials for the development of novel cancer therapies.

## Conclusion

The role and impact of modulation of p53 in regulation of autophagy is complex and far from fully clarified. Emerging evidence and rapidly developed omics as well as genome editing techniques have likely been to revolutionized a new p53 roles in autophagic activities of different p53 proteins may vary along with changes in tumor microenvironment. Therefore, novel technologies may shed a new perceptions for a knowledge-based insights to recognize gaps-existing knowledge in addition to analyze scenarios which involve a reconsideration for the function of p53 modulation in autophagy signaling in cancer. Recently, autophagy has been established as a dual role in tumor suppression process likely involved in human cancer research. p53 might be an essential player in the modulation of autophagy pathway, although the exact molecular mechanisms and cellular function in cytoplasmic and nuclear p53-mediated autophagy regulation have not been well studied. However, cellular function and role of p53-mediated autophagy, as well as molecular metabolism in cancer progression, require a strongly related and rapidly altering field. The regulation of cancer metabolism by p53 target genes can diverge according to the stress signal, cell type, and other conditions. Additional, it is evidently established that p53 stabilization is a tumor-specific vulnerability, approaches to indorse the degradation of p53 through autophagy which represents an attractive anti-cancer method. Nevertheless, our augmented understanding of the function of p53 and autophagy will hopefully offer a prospective approach to cancer treatment. Therefore, this review revealed that p53 could be targeted as an important implication of cancer therapy via modulation of autophagy signaling. Hitherto the actual therapeutic use of p53-mediated autophagy induction needs detailed knowledge of how the autophagy-lysosomal pathway may affect in cancer progression.
